# P225: Use alcohol swab in the prevention of bloodstream infections associated with catheters in the intensive care unit of a hospital infectious disease

**DOI:** 10.1186/2047-2994-2-S1-P225

**Published:** 2013-06-20

**Authors:** ACDS Oliveira, S Scota, AM Costa, E Silva, M Costa

**Affiliations:** 1 Educação Continuada e CCIH, Instituto de Infectologia Emílio Ribas, São Paulo, Brazil

## Introduction

The primary bloodstream infections are among the most common related to health care. It is estimated that about 60% of nosocomial bacteremia are associated with some intravascular device. Among the most common known risk factors for infection highlights the use of central venous catheters, especially those with short tenure. The high rate of morbidity and mortality and high cost attributed to bloodstream infections, punctured the new preventive approaches and safe practices with the use of alcohol swab catheter hubs.

## Objective

To evaluate the impact of the use of alcohol swab as a method of prevention of bloodstream infections associated with catheters.

## Method

in September of 2012 was instituted the use of alcohol swab to disinfect the connections (hubs) of catheters. Was conducted classroom training and on-site nursing staff of ICU, encouraging the proper use of the product.

## Results

The mean density of bloodstream infection in 2012 was 13.52 per 1000 catheter/day. In september when he began using alcohol swab and training, the density was 19.23 per 1000 catheter/day. A month after using the swab was sharp fall in infections, passing the 4.42 per 1000 catheter/day. In november the rate of bloodstream infection was 7.30 per 1000 catheter/day. In december after three months of training the density of infection ascended, reached 18.40 per 1000 catheter/day.

## Conclusions

can infer that the use of alcohol swab and continuous training of safe practice is the best weapon to combat bloodstream infection.

## Disclosure of interest

None declared

